# Nitrous oxide (N_2_O) emission characteristics of farmland (rice, wheat, and maize) based on different fertilization strategies

**DOI:** 10.1371/journal.pone.0305385

**Published:** 2024-07-08

**Authors:** Dingmu Hou, Xuanchen Meng, Mengting Qin, Ennan Zheng, Peng Chen, Fanxiang Meng, Chao Zhang

**Affiliations:** 1 School of Hydraulic and Electric Power, Heilongjiang University, Harbin, China; 2 College of Hydrology and Water Resources, Hohai University, Nanjing, China; 3 College of Hydraulic Engineering, Zhejiang Tongji Vocational College of Science and Technology, Zhejiang, China; Center for Research and Technology Transfer, VIET NAM

## Abstract

Fertilizer application is the basis for ensuring high yield, high quality and high efficiency of farmland. In order to meet the demand for food with the increasing of population, the application of nitrogen fertilizer will be further increased, which will lead to problems such as N_2_O emission and nitrogen loss from farmland, it will easily deteriorate the soil and water environment of farmland, and will not conducive to the sustainable development of modern agriculture. However, optimizing fertilizer management is an important way to solve this problem. While, due to the differences in the study conditions (geographical location, environmental conditions, experimental design, etc.), leading to the results obtained in the literatures about the N_2_O emission with different nitrogen fertilizer application strategies have significant differences, which requiring further comprehensive quantitative analysis. Therefore, we analyzed the effects of nitrogen fertilizer application strategies (different fertilizer types and fertilizer application rates) on N_2_O emissions from the fields (rice, wheat and maize) based on the Meta-analysis using 67 published studies (including 1289 comparisons). For the three crops, inorganic fertilizer application significantly increased on-farm N_2_O emissions by 19.7–101.05% for all three; and organic fertilizer increased N_2_O emissions by 28.16% and 69.44% in wheat and maize fields, respectively, but the application of organic fertilizer in rice field significantly reduced N_2_O emissions by 58.1%. The results showed that overall, the application of inorganic fertilizers resulted in higher N_2_O emissions from farmland compared to the application of organic fertilizers. In addition, in this study, the average annual temperature, annual precipitation, soil type, pH, soil total nitrogen content, soil organic carbon content, and soil bulk weight were used as the main influencing factors of N_2_O emission under nitrogen fertilizer strategies, and the results of the study can provide a reference for the development of integrated management measures to control greenhouse gas emissions from agricultural soils.

## Introduction

Nitrous oxide (N_2_O), a significant greenhouse gas (GHG) following carbon dioxide (CO_2_) and methane (CH_4_), contributes 6–10% to global warming [1]. It possesses a global warming potential (GWP) 265 times greater than CO_2_ [2]. Notably, agricultural land is responsible for 60% of total global anthropogenic N_2_O emissions [3]. According to the Greenhouse Gas Bulletin published by the World Meteorological Organization (WMO) in 2023, N_2_O levels experienced the most considerable year-on-year increase between 2021 and 2022, signifying a critical environmental concern.

Agricultural land, as the largest anthropogenic N_2_O sources [4, 5], have seen a 30% rise in emissions over the last four decade. This surge, primarily attributed to increased nitrogen fertilizer usage, is a primary driver of rising atmospheric N_2_O levels. Global food demand is projected to grow between 100 and 110 percent by 2050 [6], with a consequent dramatic increase in global nitrogen fertilizer application, which will exceed 1.86×108 Mg N yr^-1^ [7]. Consequently, N_2_O emissions from agricultural soils may reach 6–7 Tg N/year by 2030, driven by this heightened demand [8]. The Sixth Synthesis Report of the IPCC emphasizes the adverse impact of climate change on agricultural productivity (IPCC2023). The excessive application of nitrogen fertilizers on farmlands leads to substantial nitrogen retention in the soil. This excess nitrogen often leaches into water bodies or is released as N_2_O [9] during or after crop growth seasons [10], causing environmental challenges such as water quality degradation and increased atmospheric N_2_O concentrations [11]. In order to alleviate these problems, at present, there are many greenhouse gas emission reduction measures: for example, intermittent irrigation [12], which advocates fertilizer and water-saving irrigation techniques that can realize fertilizer and water integration, can achieve scientific water supply according to the different fertility periods of crops and different seasons, can reduce water and fertilizer loss, and optimize the growing environment of crops. And among them, optimizing fertilizer application is vital. Methods like coordinated (organic and inorganic) and rational (crop-specific) fertilizer applications can significantly reduce greenhouse gas emissions from agricultural cultivation [12]. While irrational nitrogen fertilizer usage causes severe environmental pollution, rational application supports soil fertility, enhances crop yields, and curtails N_2_O emissions. This optimization plays a crucial role in improving crop nutrient use efficiency, yield, and reducing N_2_O emissions [13]. In regions like sub-Saharan Africa, the underuse of inorganic fertilizers leads to nutrient depletion and land degradation, diminishing crop yields. Conversely, adopting crop systems and nitrogen fertilizers suited to local conditions can replenish soil nitrogen [14].

The type and quantity of fertilizer applied significantly influence N_2_O emissions [15]. In Asia and Africa, organic and inorganic fertilizers are widely used to sustain agricultural production. Moreover, Traditional organic fertilizers, such as livestock manure, not only reduce the total nitrogen input [16] but also facilitate resource recycling when applied to farmlands [17], which can lead to nitrogen loss. Organic manure enhances soil matter cycling, providing essential nutrients for crop growth and maturation [18]. Lehtinen *et al*. [19] reported the quantitative effect of applied organic fertilizer on greenhouse gas emissions, which resulted in a 12-fold increase in N_2_O emissions compared to a control trial without organic fertilizer, promoting soil organic carbon (SOC) accumulation [20]. Agricultural crops are mainly dominated by inorganic nitrogen uptake [21]. However, the water solubility of most inorganic fertilizers leads to heightened nitrogen loss, affecting crop nitrogen utilization [22]. Hayatu, N.G. *et al*. mentioned that the co-application of organic and inorganic fertilizers can achieve a long-term supply of soil nutrients to ensure the growth of crops [23]. Comprehensive existing studies show that the research on N_2_O emission from farmland by organic and inorganic fertilizers has been increasingly concerned by experts and scholars.

In subtropical Asia, the rice-wheat cropping system occupies 26 Mha of arable land, with 105,000 ha of arable land devoted to rice and wheat in the Indian region alone [24], and agricultural GHG emissions account for 16 percent of the country’s total GHG emissions [25]. Rice, wheat, and maize are globally pivotal food crops, with rice being notably more GHG-intensive than wheat and maize according to China’s National Bureau of Statistics [26]. Zhang *et al*. [26] assessed the contribution of wheat to N_2_O emissions and showed that on-farm N_2_O emissions are strongly influenced by crops. The results obtained by Smart *et al*. [27] showed that wheat releases N_2_O during the uptake of assimilated nitrate nitrogen. Zhang *et al*. [18] found that nitrification proceeded slower in the summer maize season in an acidic soil and that the N_2_O emission flux was significantly higher than that in neutral and alkaline soils. N_2_O emission fluxes were significantly higher than those of neutral and alkaline soils. Cui et al’s [28] analysis revealed a positive correlation between maize seasonal N_2_O emission and soil organic carbon content. It can be seen that factors such as different crops and different soil physicochemical properties affect the N_2_O emission from farmland [29]. A meta-analysis by Niu *et al*. [30] found that previous studies [31] have yielded a 50% reduction in N_2_O emissions during the wheat season, and a 37% reduction during the maize season, with the latter’s lower emissions being attributed to a lower rate of nitrification. Therefore, it is possible to reduce N_2_O emissions by slowing down nitrification, improving nitrogen use efficiency and reducing subsequent denitrification [32]. Many studies on N_2_O emissions from production systems were mentioned earlier, but Ling Lin *et al*. [12] found few reports on on-farm production systems with minimal GHG emissions but high economic returns. Among the literature we chose to study, the importance of including socio-economic factors in relevant studies was affirmed by Zhen Liu *et al*. [33] who argued that soil quality and crop yields can still be maintained at high levels by reducing N application, while reducing costs and protecting the environment. Therefore, how to effectively reduce N_2_O emissions from farmland while ensuring economic benefits is also an issue we need to address. Socio-economic factors are considered to ensure the consistency between research results and practical applications, and to ensure that the recommended fertilization strategies are not only scientifically sound, but also economically feasible, and can be widely accepted and implemented by the society.

Numerous field measurements have been conducted to examine N_2_O emissions triggered by nitrogen fertilizer use, analyzing influencing factors such as soil physicochemical properties, nitrogen fertilizer amounts, and crop types [34]. The variability of factors controlling N_2_O production significantly increases the uncertainty in emission estimates [35], with only a few studies thoroughly examining these explanatory factors [36]. A quantitative understanding of the potential linkages between N_2_O emissions and their influencing factors is crucial for effectively implementing strategies that address both food security and climate change mitigation [37]. Although many experiments have been conducted to measure the relevance of N fertilizer in influencing N_2_O emissions, these have produced different results in different study areas due to factors such as geography, soil type and field management, and it is difficult to draw general conclusions without quantitative analysis. N. BORDOLOI [38] mentioned that the impact of nitrogen fertilizer management on N_2_O emissions from Indian agriculture is under-reported. Most of the studies on N_2_O emissions from crops have examined only a single nitrogen fertilizer input and lacked controlled experiments [39], and these studies could not fully measure the relationship that exists between nitrogen inputs and N_2_O emissions [40]. In this study, Meta-analysis was used in conjunction with the results of collected field trial data to quantify the effect of different fertilization practices on the correlation of N_2_O emissions. In this study, Meta-analysis was used in conjunction with the results of collected field trial data to quantify the effect of different fertilization practices on the correlation of N_2_O emissions, thus contributing to a nuanced understanding of on-farm N_2_O emissions across three major crop systems and their response to varied nitrogen fertilizer applications. The main objectives of this study were to (1) Assess the impact of varied fertilizer application strategies on N emissions in agricultural soils; (2) Identify and analyze critical determinants of N emissions following fertilizer application, evaluate the influence of different nitrogen fertilizer types and application quantities on N_2_O emissions; and (3) Investigate and elucidate the underlying factors influencing N_2_O emission variations, exploring effective fertilizer application regimes that could potentially reduce these emissions.

## Materials and methods

### Data collection

This study used N_2_O emissions as a response to a set of fertilizer management options. Only studies that reported a specific experimental design with a control trial were used. For instance, comparisons were drawn between treatments with and without N fertilizer application, under otherwise constant conditions. Trials were categorized based on fertilizer application, fertilizer types, and crop species to identify key influencing factors.

In this study, we searched for relevant papers published in peer-reviewed journals between 2000 and 2023 using Web of Science, ELSEVIER ScienceDirect, and the China Knowledge Resources Integrated Database (CNKI). Keywords such as: “nitrogen emission”, “nitrogen fertilizer”, “N_2_O emission”, “field crop”, “rice”, “wheat” and “maize” were employed in the research. Extracted data included N_2_O emissions, soil ammonium nitrogen (NH_4_^+^), nitrate nitrogen (NO_3_^-^), and average soil organic carbon (SOC) concentrations across various treatments. Studies were selected based on the following criteria: (1) on-farm studies of rice, wheat, or maize growth in each region provided detailed information on experimental location, design, and conditions; (2) sample means were reported for both the control with no N fertilizer and the treatments with applied organic or inorganic fertilizers; (3) at least three replications were included; (4) data on at least one of the target variables (nitrogen use efficiency, N_2_O emission flux, SOC content, soil ammonium N and nitrate N content) were quantified and cumulative data were reported for at least one growing period.

From this methodology, 67 papers were selected encompassing 23 (132 comparisons) for organic fertilizers, 61 (424 comparisons) for inorganic fertilizers; 29 (175 comparisons) were related to rice, 38 (274 comparisons) were related to wheat and 31 (284 comparisons) were related to maize. Among them, the information included in the study includes geographical location of the study area, soil physicochemical properties (soil type, soil pH, total nitrogen content, and soil bulk weight), climatic conditions (mean annual temperature and mean annual precipitation), crop type (rice, wheat, and maize), the average cumulative N_2_O emissions, standard deviation (SD), number of replicates, inorganic fertilizers (general nitrogen fertilizer and urea), organic fertilizers (animal manure and compost), and the average cumulative N_2_O emissions were as follows and fertilizer application, used to quantify statistical relationships of N_2_O emissions. All data were extracted from the articles, and graphical data were digitized using GetData Graph Digitizer 2.24.

### Evaluated variables and grouping

**Fig 1:**
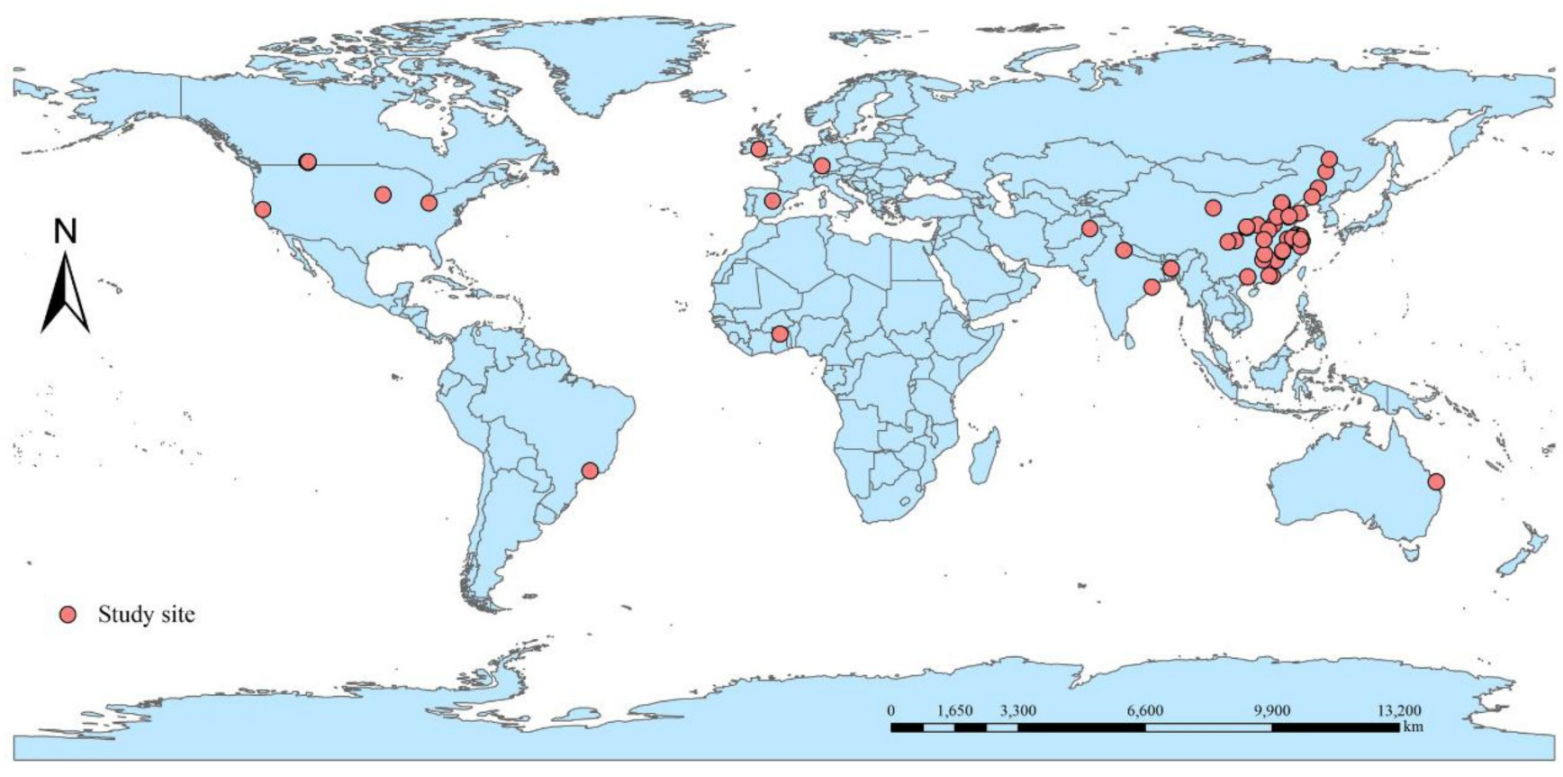
Overview map of the global study area.

In this research, geographic coordinates (latitude and longitude) were recorded for 67 study areas, leading to the creation of a comprehensive global study area profile, as depicted in Fig 1. The dataset, comprising results from 66 distinct locations, encompasses contributions from over ten countries, including China, the United States, and India. Analysis of the study locations’ distribution reveals a significant concentration in Asia (80.3%), followed by 13.64% in the Americas, with the remaining 6.06% spread across other continents.

After screening based on the selected criteria, we extracted the mean, standard deviation (SD) and sample size in each study. In instances where the SD was not provided, we utilized the standard error (SE) to calculate it using Eq (1), where n denotes the number of replications (sample size). For cases lacking both SD and SE values, a regression equation was formulated by linearly fitting SD data with other corresponding mean values from the same study, enabling us to estimate the required SD. In scenarios where pertinent data for these calculations were absent, we assigned SE as 1/10th of the mean value, as per standard practice in statistical analysis [3], and the required data were then calculated.


$$\text{SD}=\text{SE}\times \sqrt{\text{n}}$$ (1)

We divided each explanatory variable into different groups due to the different field management practices used in the selected experimental farmland. According to the profile of the selected study area, based on the recommendations given by Lou *et al*. [41]: the mean annual temperature (MAT) was classified into three categories (<15°C; 15–20°C; ≥20°C), and the mean annual precipitation (MAP) was classified into three categories (<600mm; 600-1000mm; ≥1000mm). Nitrogen application were categorized as low (<150 kg N ha^-1^), medium (150–225 kg N ha^-1^) and high (≥225 kg N ha^-1^). Soil types were divided into sandy, loamy, clayey and anthropogenic categories. Soil pH levels were categorized into acidic soils (≤6.5), neutral (6.5–7.5), and alkaline (≥7.5), while organic carbon (OC) was categorized into three levels (<8 g C kg^-1^; 8–12 g C kg^-1^; ≥12 g C kg^-1^) [42]. Soil total nitrogen (TN) content was categorized into three groups (<0.9 g N kg^-1^; 0.9–1.4 g N kg^-1^; ≥1.4 g N kg^-1^) and soil bulk density (BD) data were categorized into three groups (<1.2 g cm^-3^; 1.2–1.4 g cm^-3^; ≥1.4 g cm^-3^).

This study focused on the effects of nitrogen fertilization on crop N losses and soil N dynamics. (1) N losses (including N_2_O emissions); and (2) soil physicochemical properties of soil layers (TN, SOC, NH_4_^+^, NO_3_^-^, and soil pH), prioritizing the 0–20 cm soil layer when multiple layers were present. This investigation aims to elucidate the interactions between N losses and influencing factors, enhancing our understanding of fertilization’s overall impact on soil N emissions.

### Data analysis

In this study, effect sizes were expressed as the natural logarithm of the response ratio (RR) [43], calculated according to Eqs (2) and (3):

$$\text{RR}=\frac{{\text{X}}_{\text{t}}}{{\text{X}}_{\text{c}}}$$ (2)

$$\text{ln}\;\text{RR}=\text{ln}\left(\frac{{\text{X}}_{\text{t}}}{{\text{X}}_{\text{c}}}\right)=\text{ln}\;{\text{X}}_{\text{t}}-\text{ln}\;{\text{X}}_{\text{c}}$$ (3)

Where Xt is the treatment group (nitrogen application) and X_c_ is the control group (no nitrogen application). The variance (v) of each RR was calculated using Eq (4) [44]:

$$\text{v}=\frac{{\text{S}}_{\text{t}}^{2}}{{\text{n}}_{\text{t}}{\text{x}}_{\text{t}}^{2}}+\frac{{\text{S}}_{\text{c}}^{2}}{{\text{n}}_{\text{c}}{\text{x}}_{\text{c}}^{2}}$$ (4)

where n_t_ and n_c_ denote the sample size of the treatment and control groups, respectively, and “St” and “Sc” denote the SD of the treatment and control groups, respectively.

Weighted effect sizes and 95% confidence intervals (CIs) were calculated using a random effects model based on restricted maximum likelihood estimation (RMLE) with OpenMEE software [43]. Heterogeneity between samples was calculated using the cumulative effects model, and inter-case variability was derived using I2 to synthesize the results for significance. All data analysis and graphs were performed using OriginPro 2022 (OriginLab Corporation, Massachusetts, USA).

### Analysis of publication bias

Fail-safe coefficients (Rosenberg’s Nfs) were used to indicate the possible presence of publication bias [45, 46], where a fail-safe number greater than (5n+10) indicates the absence of publication bias. The results showed that:

The number of failures of organic and inorganic fertilizer application on N_2_O emission were 301333 and 22158855, respectively. These fail-safe numbers were much larger than the number of comparisons in our dataset, thus indicating that our results were not affected by publication bias and the effect values obtained were reliable.

## Results

### Response of N_2_O emissions to different fertilization methods

**Fig 2:**
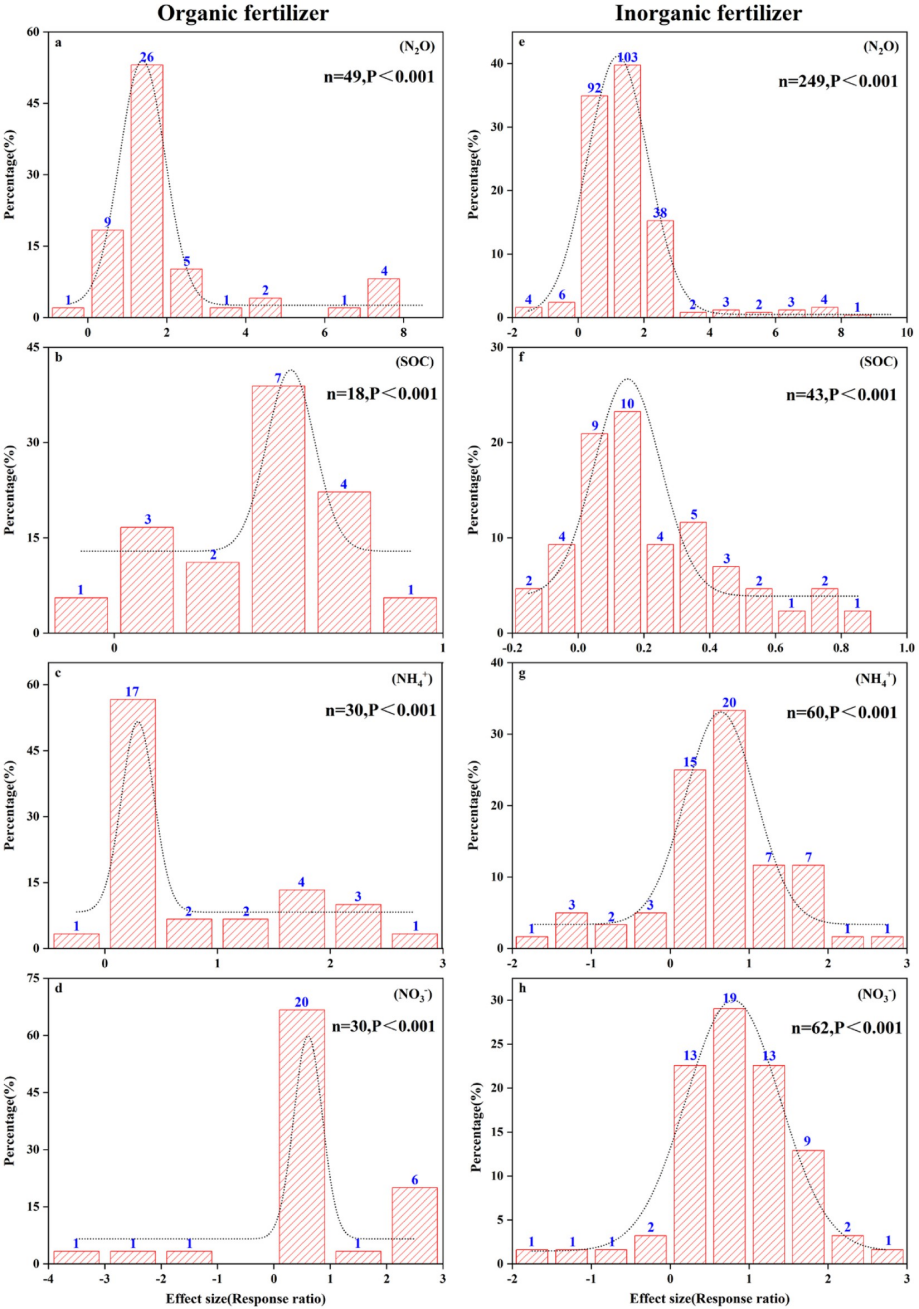
Frequency distribution (a-h) of the effect values (response ratios) of different fertilization practices on various soil physical and chemical properties. “n” represents the sample size, and “p” represents the significance level. The dashed line shows the normal distribution curve of the effect values.

**Fig 3:**
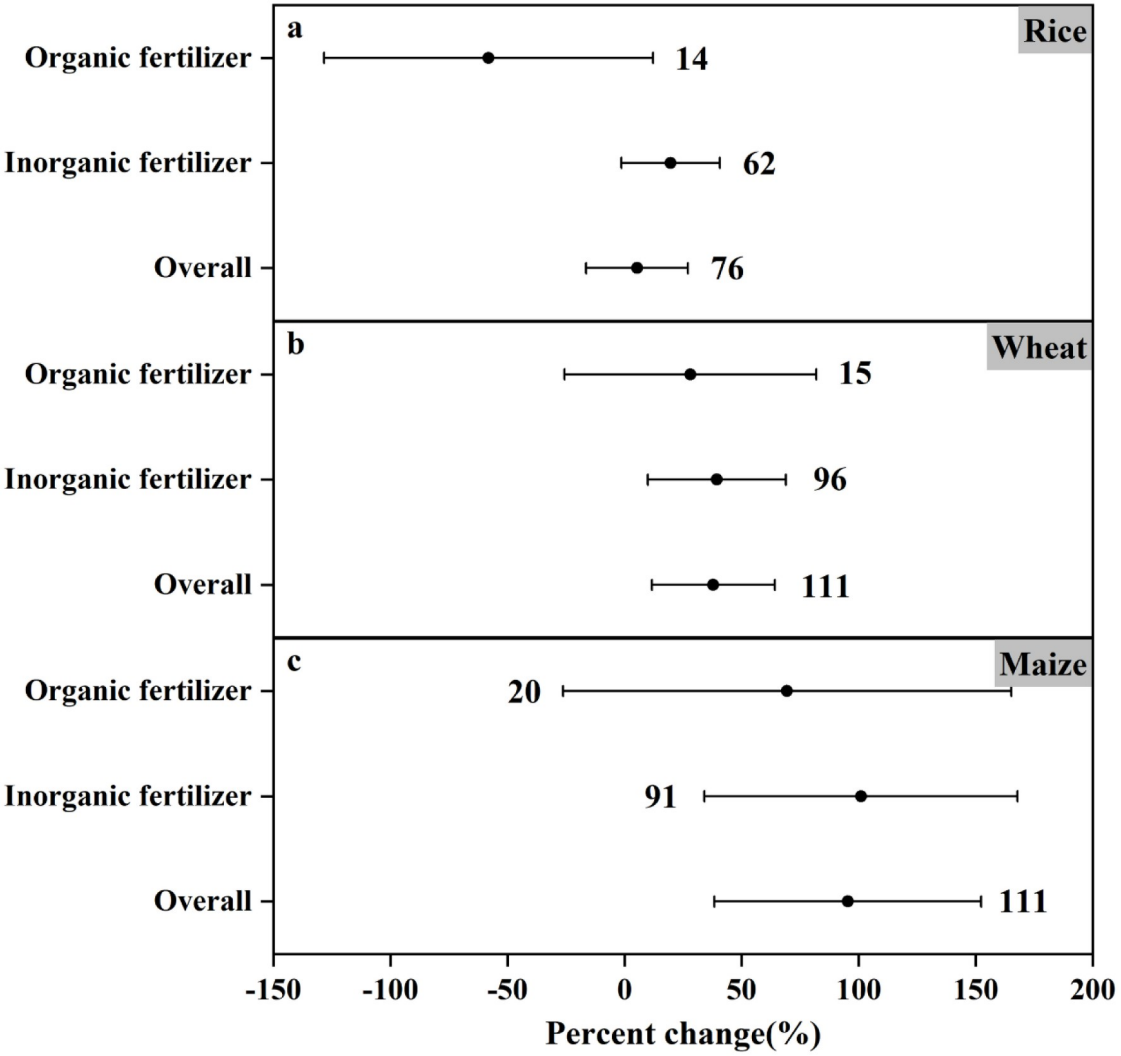
Comparison of N_2_O emissions from different fertilization practices with major crop types (a-c).

Our analysis revealed that the logarithmic response ratios (ln(RR)) of nitrogen emission factors under various fertilization methods followed a Gaussian normal distribution (Fig 2a–2h). This pattern suggests homogeneity within the dataset [42]. N_2_O production, a key route of soil N emission, varied with fertilization method and crop soil type, as illustrated in Fig 3a–3c. The application of inorganic fertilizers significantly increased N_2_O emissions from agricultural fields (19.70% increase in paddy fields, 39.41% increase in wheat fields, and 101.05% increase in maize fields), while the application of organic fertilizers contributed to N_2_O emissions in both wheat fields (28.16% increase) and maize fields (69.44% increase), but in paddy fields N_2_O emissions were significantly reduced by 58.10%. It can be seen that both fertilizer application methods and crop soil types have significant effects on N_2_O emissions from farmland. Therefore, different crop soil types can be used to control fertilizer application, and reasonable fertilizer application methods with significant economic benefits and environmental friendliness can be sought to reduce farmland N_2_O emissions.

### Response of N_2_O emissions to fertilizer types under different experimental conditions

**Fig 4:**
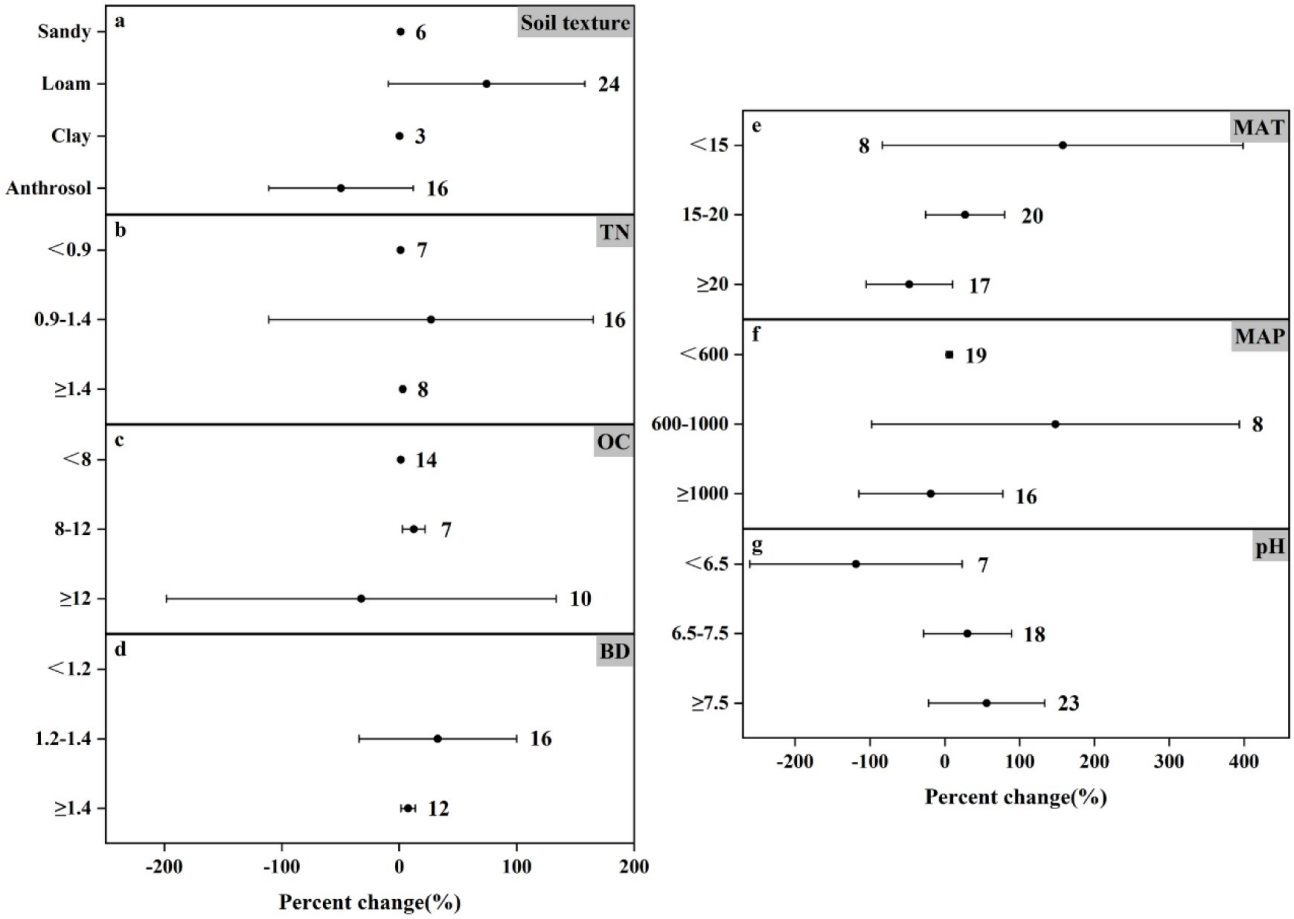
Effects of organic fertilizer application on changes in N_2_O emissions (%) in different experimental environments. Dots indicate mean effects, numbers indicate sample size, and error bars indicate 95% confidence intervals (CI).

**Fig 5:**
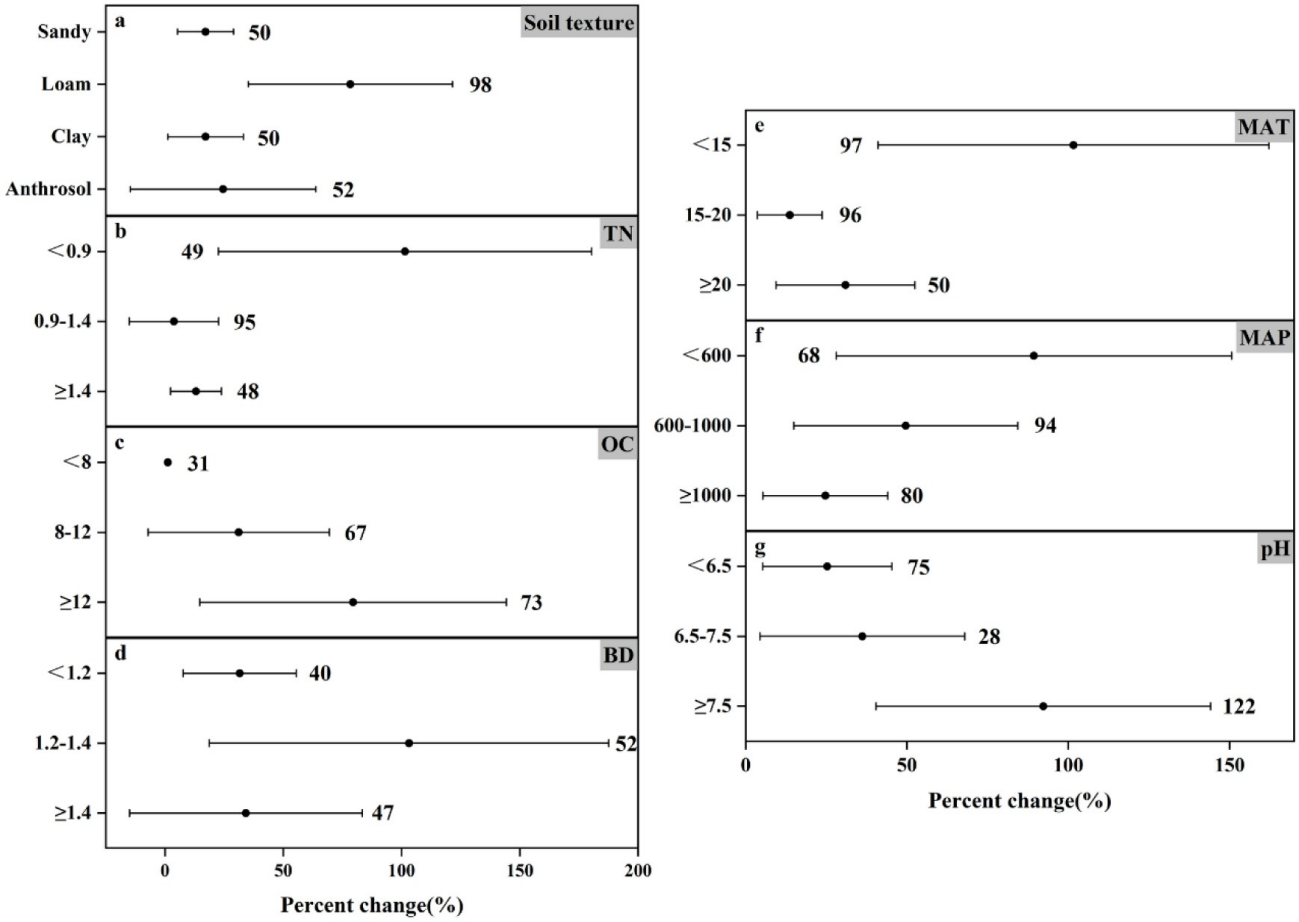
Effect of applied inorganic fertilizer on changes in N_2_O emissions (%) in different experimental environments. Dots indicate mean effects, numbers indicate sample size, and error bars indicate 95% confidence intervals (CI).

The response of N_2_O emissions to different fertilization types varies under different influencing factors (Figs 4 and 5). Climate factors, soil properties, and nitrogen application levels played crucial roles. For climate factors MAT and MAP, the two fertilizer types affected N_2_O emission differently. At MAT<15°C, organic and inorganic fertilizers increased N_2_O emission by 157.63% and 101.62%, respectively, which were more significant; at MAT of 15–20°C, they increased N_2_O emission by 27.05% and 13.67%, respectively; at MAT≥20°C, inorganic fertilizers increased N_2_O emission by 30.93% while organic fertilizers N_2_O emission by 47.47%. The effect from precipitation was equally important. At MAP<600 mm, organic and inorganic fertilizers increased N_2_O emission by 5.89% and 89.34%, respectively; at MAP of 600–1000 mm, they increased N_2_O emission by 147.89% and 49.62%, respectively; the difference was that at MAP≥1000 mm, inorganic fertilizers increased N_2_O emission by 24.69%, whereas application of organic fertilizers but reduced N_2_O emission (by 18.83%).

Soil texture, as one of the important physical properties of soil, not only affects crop yield and nitrogen fertilizer efficiency, but also influences greenhouse gas emissions from agricultural soils [47]. Inorganic fertilizers significantly contributed to N_2_O emissions in all four soil textures (Fig 5a), The only reduction in N_2_O emissions was the application of organic fertilizers to anthropogenic soils. Organic carbon plays an important role in soil fertility, water retention and nutrient supply, etc. In the analysis of this paper, the effects of both fertilizer application methods on N_2_O emission were not significant in the ranges of OC lower than 8 g C kg^-1^ and 8–12 g C kg^-1^, but the effects were significant in the high OC environment, where organic fertilizer significantly decreased by 32.40% and inorganic fertilizer significantly increased by 79.54%.

TN is one of the important indicators of soil fertility, which directly affects the growth and development of crops. Both fertilizer types increased N_2_O emission in all three ranges of TN, but the effect results were not all significant. As can be seen from the figure, the effects of applying organic and inorganic fertilizers on N_2_O emission in the high range of TN (increased by 2.99% and 17.11%, respectively) were much smaller than those in the low range of TN (increased by 1.09% and 79.94%, respectively). Based on the effect of soil pH, inorganic fertilizers (25.29%, 36.15%, and 92.28% increase from low to high, respectively) promoted N_2_O emission in all three ranges of soil pH, unlike organic fertilizers, which increased N_2_O emission in neutral and alkaline soils (30.17% and 55.87% increase, respectively), but at soil pH <6.5, N_2_O emission was significantly reduced by 118.70%. In addition, inorganic fertilizer increased N_2_O emission by 31.61% at BD<1.2g cm^-3^, organic and inorganic fertilizer increased N_2_O emission by 32.75% and 103.19%, respectively, at BD of 1.2–1.4 g cm^-3^, and both of them increased N_2_O emission by 7.54% and 34.22%, respectively, at BD≥1.4 g cm^-3^. From the overall analysis results, we found that the N_2_O emission from the farmland treated with organic fertilizer was much lower than that treated with inorganic nitrogen fertilizer. It can be seen that the application of organic fertilizer can better reduce N_2_O emission.

### Response of N_2_O emissions to nitrogen fertilizer amount under different experimental conditions

**Fig 6:**
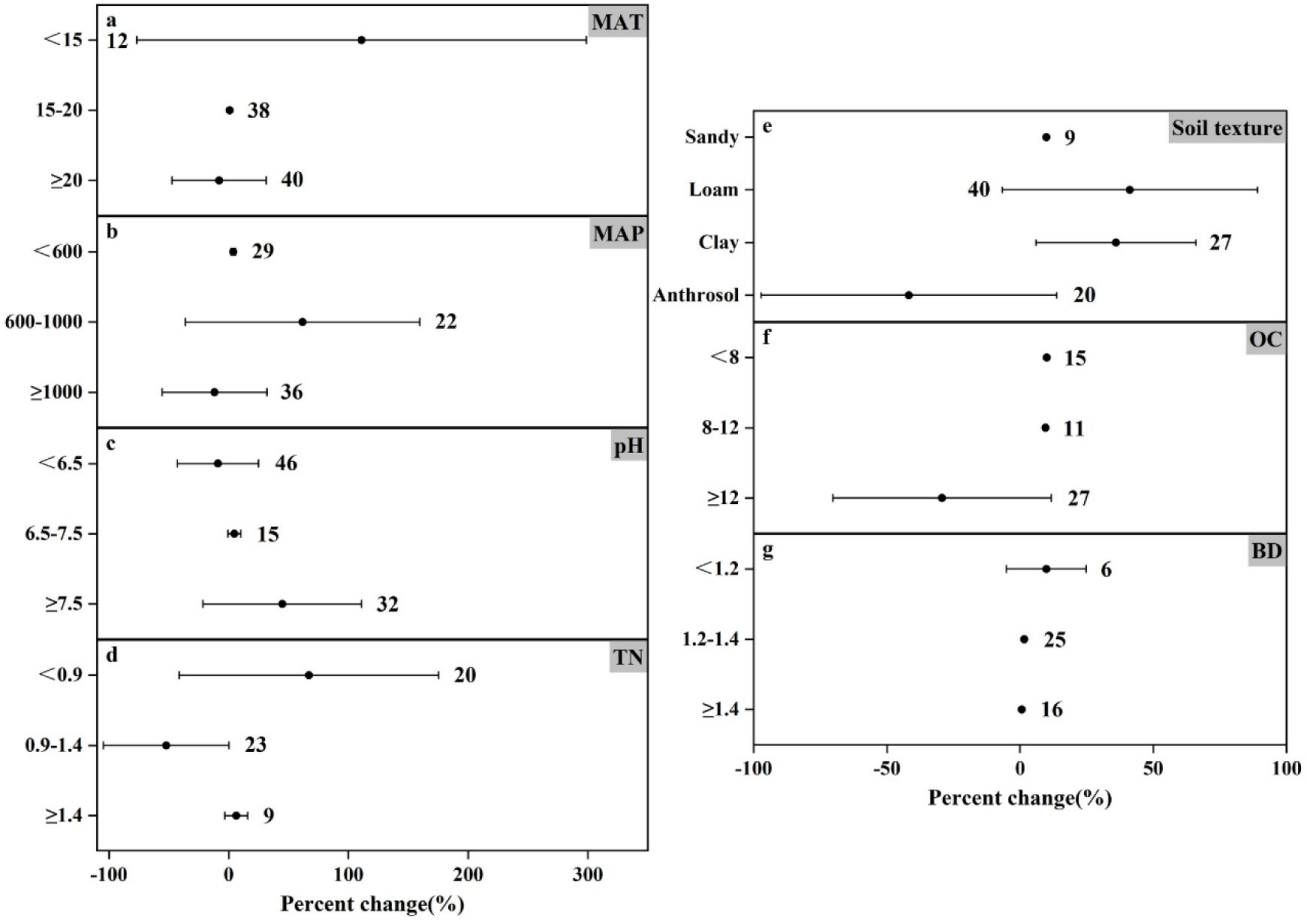
Effect of applying low amounts of nitrogen fertilizer on changes in N_2_O emissions (%) in different experimental environments. Dots indicate mean effects, numbers indicate sample size, and error bars indicate 95% confidence intervals (CI).

**Fig 7:**
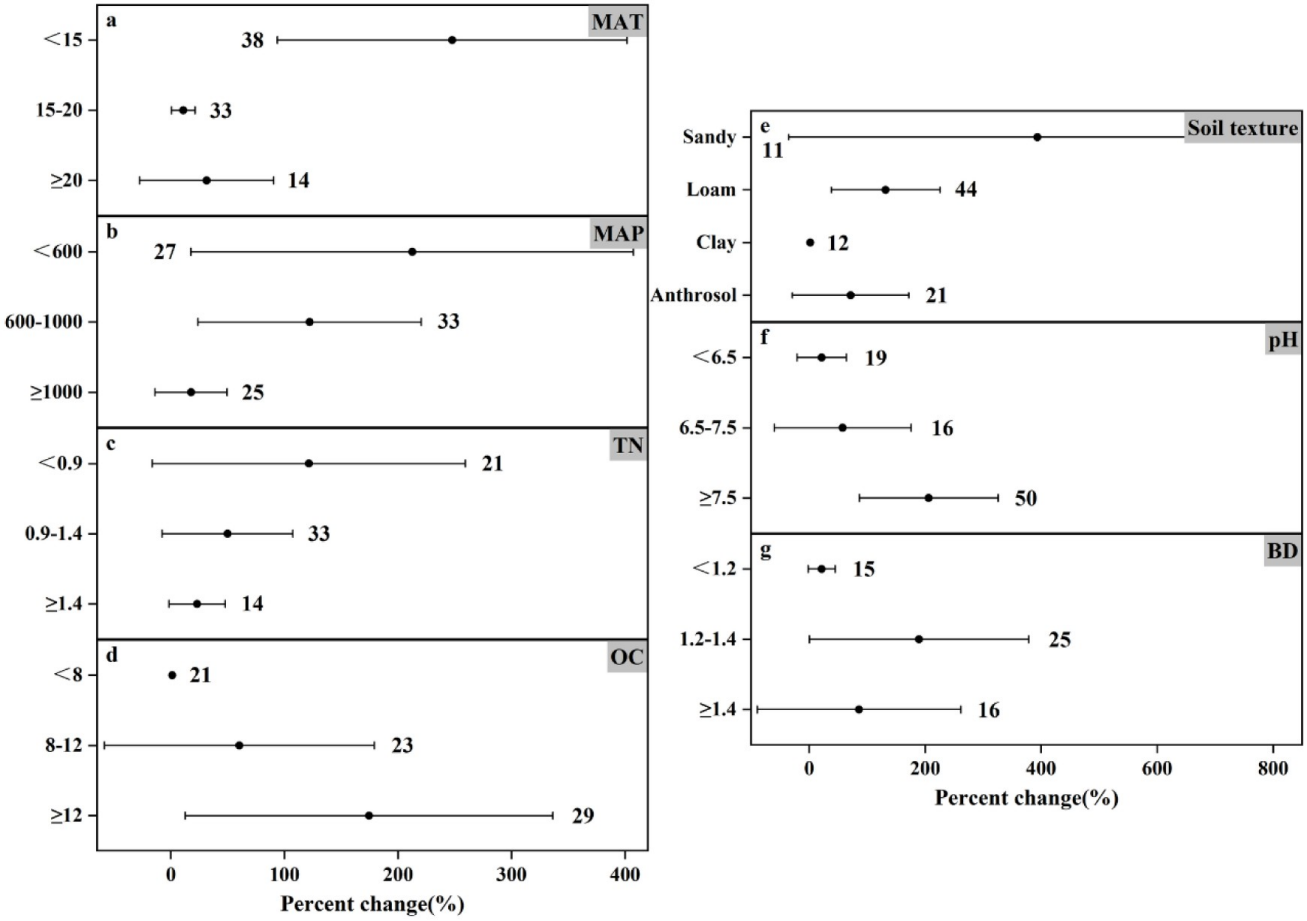
Effect of applying medium amount of nitrogen fertilizer on changes in N_2_O emissions (%) in different experimental environments. Dots indicate mean effects, numbers indicate sample sizes, and error bars indicate 95% confidence intervals (CIs).

**Fig 8:**
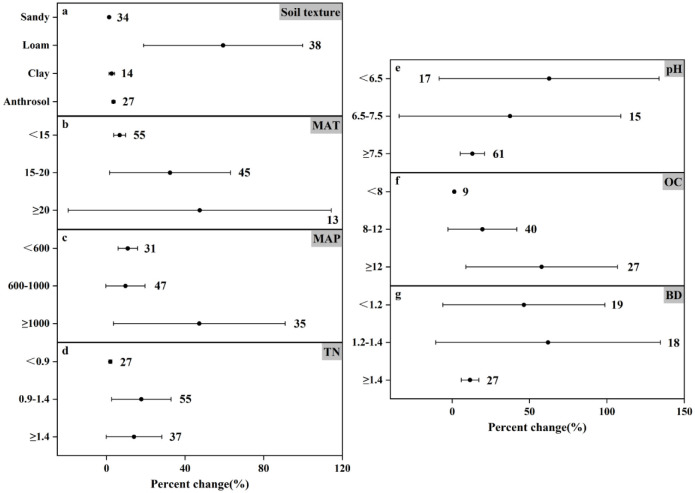
Effect of applying high amounts of nitrogen fertilizer on changes in N_2_O emissions (%) in different experimental environments. Dots indicate mean effects, numbers indicate sample sizes, and error bars indicate 95% confidence intervals (CIs).

The results of N_2_O emission under different nitrogen application rates showed (Figs 6–8) that the trends of N_2_O emission fluxes were more consistent. For different soil textures, both medium and high N application rates consistently increased N_2_O emissions and the remaining three soil types increased N_2_O emission by as much as 71.59%-393.51%, and the most significant response in high N application rate (Fig 8a) was loamy soil (increased by 59.34%), and the increase in N_2_O emission of the remaining soil types changed by less than 5%. In contrast, the effect of anthropogenic soils on N_2_O emission showed a decreasing trend (decreased by 55.92%) in the low N application (Fig 6e). In soil pH, N_2_O emission was promoted at all N application rates, except for the low N application rate, which reduced N_2_O emission (by 8.92%) at pH<6.5 (Fig 6c). Medium and high N application increased N_2_O emission at different soil TN and soil OC contents, while low N application reduced N_2_O emission (by 52.14%) at 0.9–1.4 g N kg^-1^ TN (Fig 6d) and also reduced N_2_O emission (by 42.24%) at OC≥12 g kg^-1^ (Fig 6f). In particular, all three nitrogen applications showed an increasing trend in N_2_O emission in all three categories of BD (Figs 6–8).

Temperature and precipitation significantly influenced N_2_O emissions. In low amount of nitrogen application when MAT≥20°C promoted N_2_O emission (increased by 7.98%) (Fig 6a), in temperature category where MAT was in the range of 15–20°C, it promoted N_2_O emission though not significantly but still (increased by 0.84%), and the most significant change in N_2_O emission was at MAT<15°C (increased by 111.00%). Similarly the most significant change in N_2_O emission in medium application of nitrogen was also at MAT<15°C (increased by 247.78%) (Fig 7a). The changes in N_2_O emission were promoted by both medium and high N application in all three precipitation classifications, with the difference that low N application reduced N_2_O emission (by 11.84%) at MAP≥1000mm (Fig 6b). Controlling nitrogen fertilizer inputs, rational land use, and reducing the risk posed by climate can help control N_2_O emissions.

### Response of N_2_O emissions to crop species under different experimental conditions

**Fig 9:**
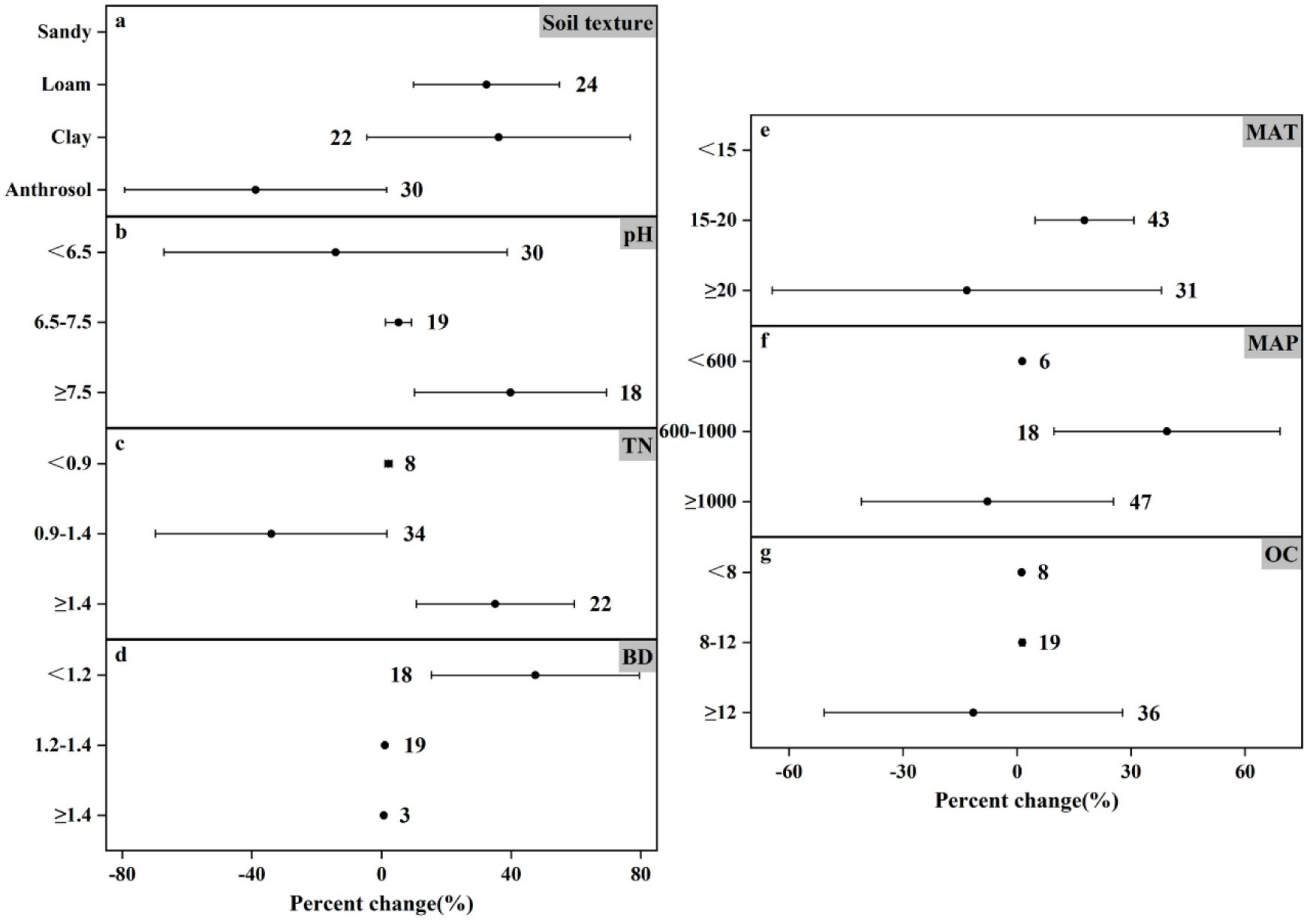
Effect of growing rice in different experimental environments on changes in N_2_O emissions (%). Dots indicate mean effects, numbers indicate sample size, and error bars indicate 95% confidence intervals (CIs).

**Fig 10:**
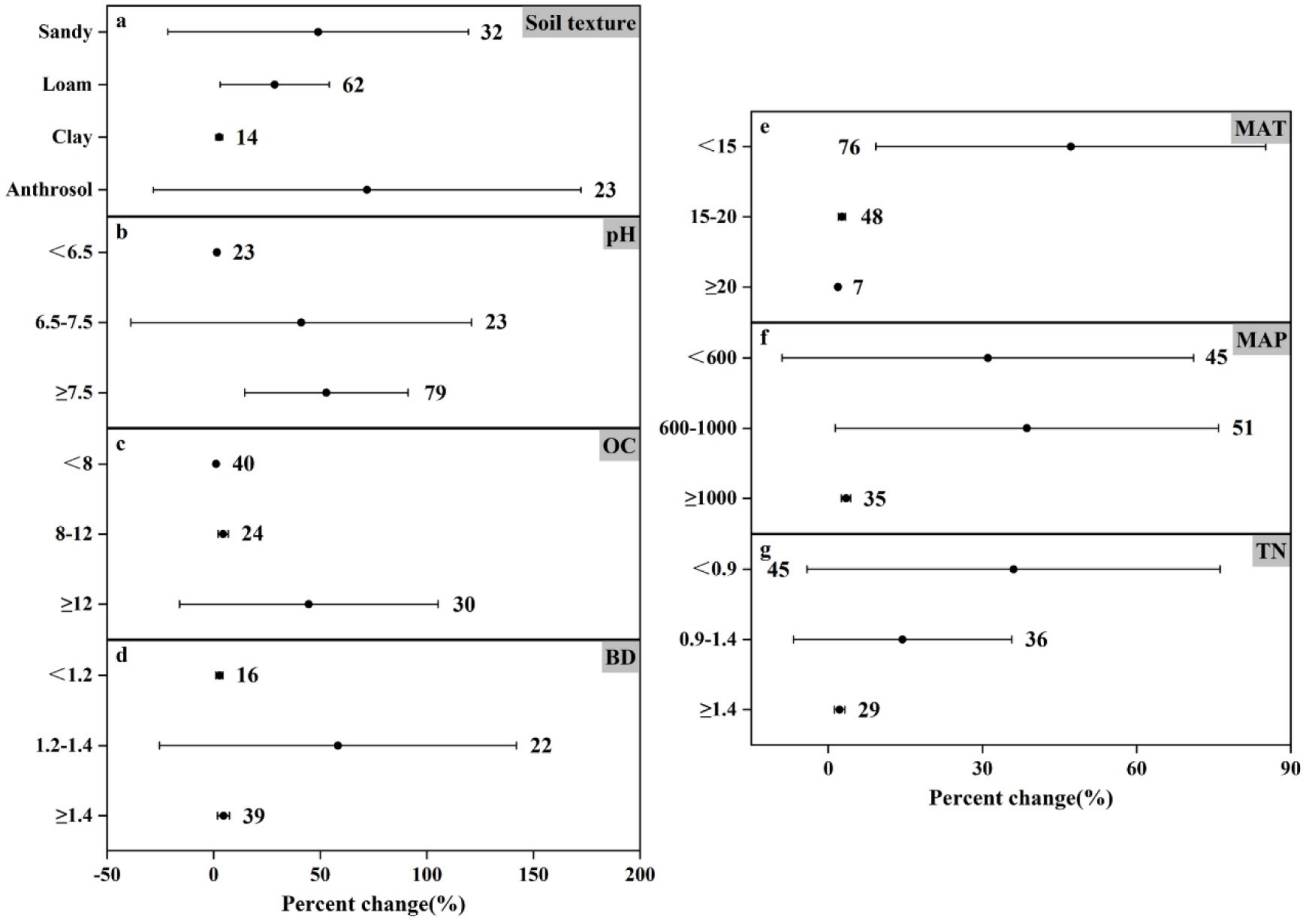
Effect of growing wheat in different experimental environments on changes in N_2_O emissions (%). Dots indicate mean effects, numbers indicate sample size, and error bars indicate 95% confidence intervals (CI).

**Fig 11:**
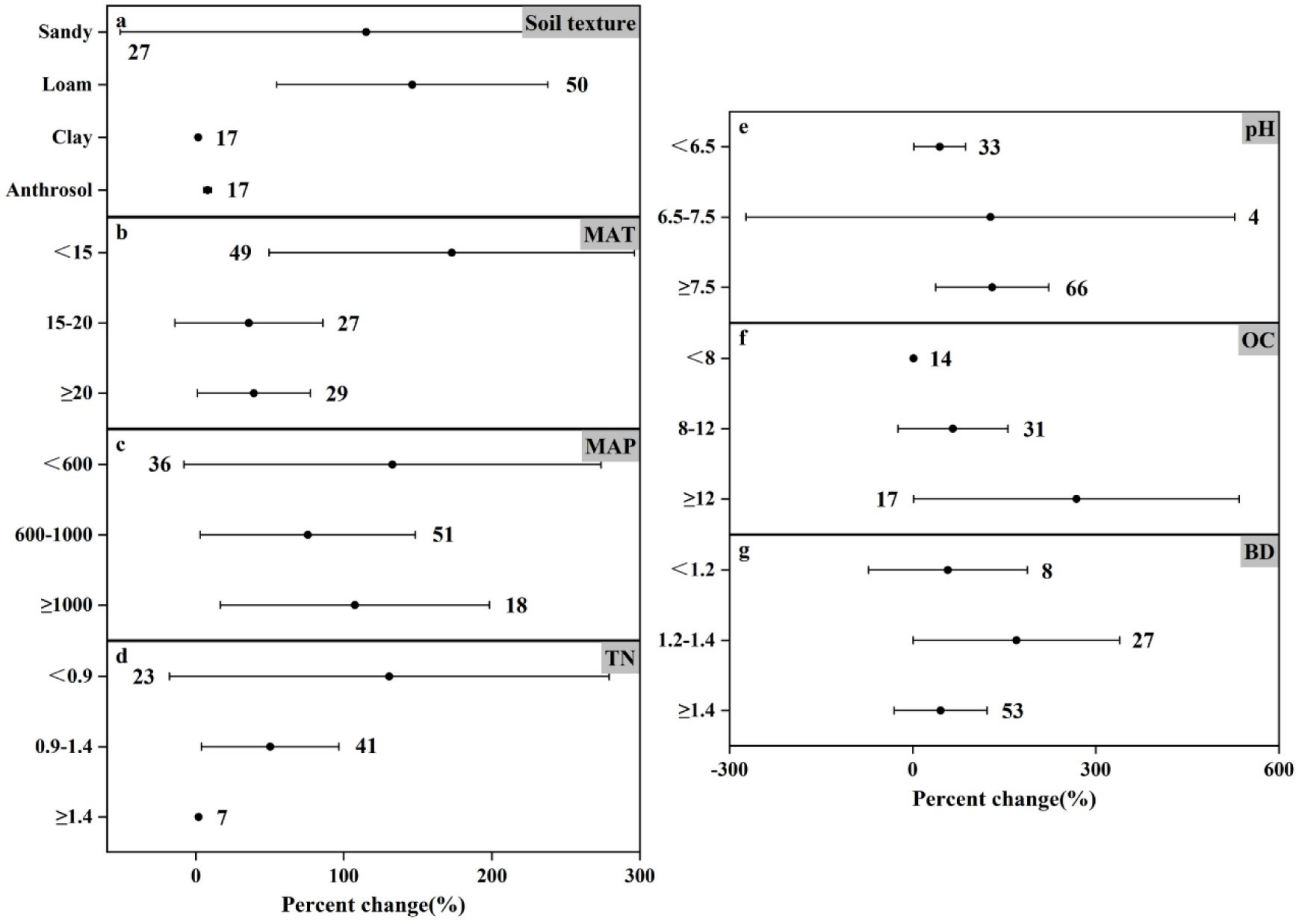
Effect of growing maize on changes in N_2_O emissions (%) in different experimental environments. Dots indicate mean effects, numbers indicate sample size, and error bars indicate 95% confidence intervals (CI).

Figs 9–11 show the effect of different crop soils on changes in N_2_O emissions. As influenced by climatic factors, rice field decreased N_2_O emission (by 13.23% and 7.81%) at higher MAT (≥20°C) and higher MAP (≥1000 mm) (Fig 9e and 9f), and wheat and maize fields were contributing to increased emissions. The most significant change in N_2_O emission was in the maize field (increased by 172.94%) when MAT<15°C (Fig 11b), and the most significant effect on the change in N_2_O emission was still in the maize field at MAP<600 mm (increased by 132.84%) under MAP conditions (Fig 11c).

Among the different soil geologic types, the most significant increase in N_2_O emission was in the maize field under sandy and loamy soil conditions (115.14% and 146.25% increase, respectively) (Fig 11a), and in the rice field under anthropogenic soil conditions, the N_2_O emission was significantly reduced by 38.84% (Fig 9a). Except that paddy field was effective in reducing N_2_O emission at pH<6.5, TN of 0.9–1.4 g N kg^-1^, and OC≥12 g C kg^-1^, the remaining three crop types planted under the two conditions of soil pH, TN, and OC promoted N_2_O emission. In addition, all three crop types, regardless of soil bulk density, showed an upward trend in N_2_O emissions (Figs 9d, 10d and 11g). These findings highlight the importance of considering crop types in managing N_2_O emissions, with specific attention to the interaction between crop cultivation and environmental conditions.

### Analysis of factors influencing N_2_O emissions

In agroecosystems, the most powerful controlling factors affecting the level of N_2_O emissions include soil nitrogen and soil carbon content [48, 49]. Correlations between the N_2_O fluxes and environmental factors were analyzed using Pearson correlation coefficients at a 0.05, 0.01 or 0.001 probability level.

**Fig 12:**
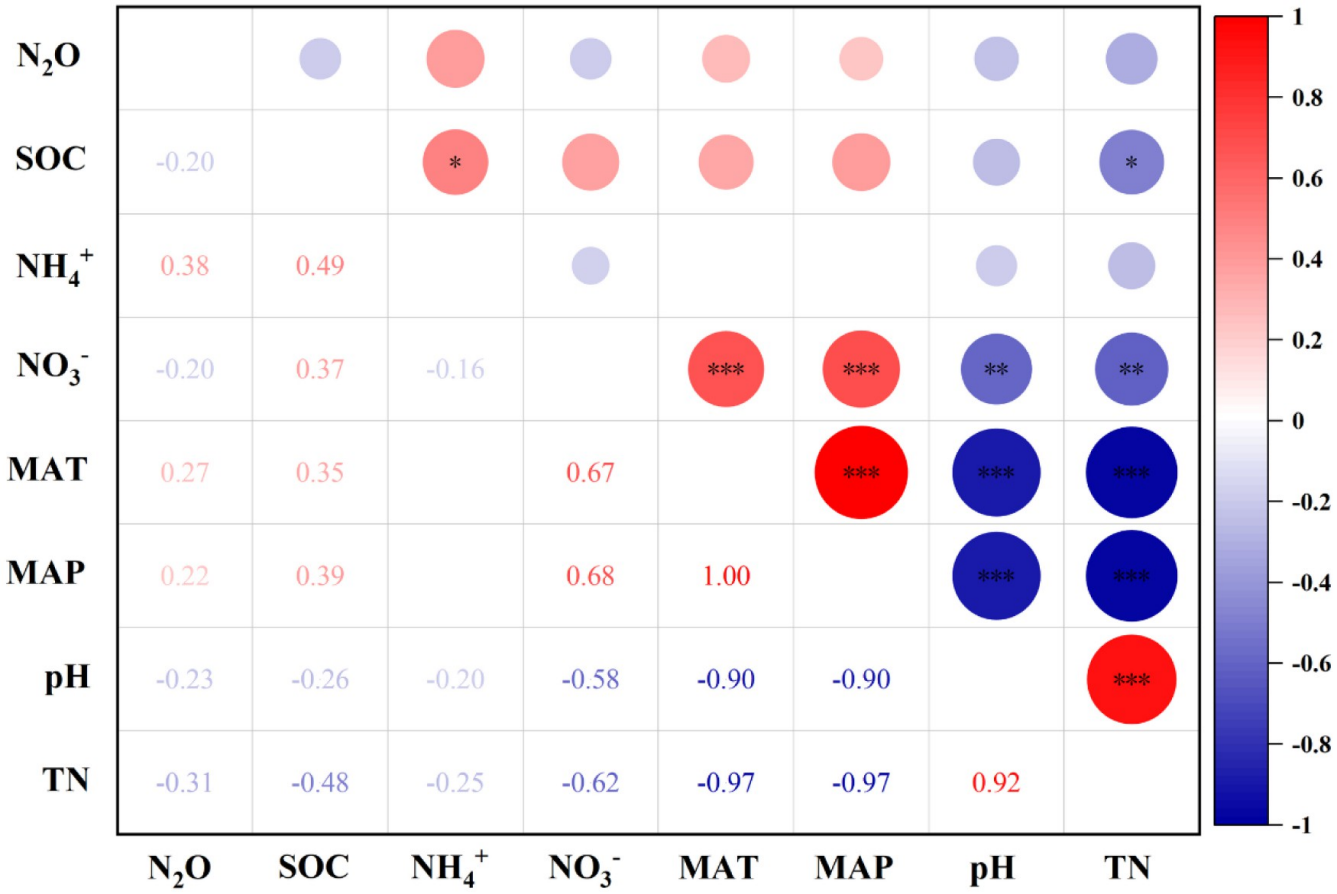
Correlation analysis of environmental impact factors and N_2_O emission in farmland. Note: *-p<0.05; **- p<0.01; ***-p<0.001.

The results of Pearson analysis showed that N_2_O emissions were positively correlated with soil NH_4_^+^ content, MAT and MAP, and negatively correlated with SOC, soil NO_3_^-^ content, soil pH and TN (Fig 12). Soil NH_4_^+^ content was negatively correlated with NO_3_^-^ content, pH and TN and positively correlated with SOC. It can be seen that the influence of climate factors on N_2_O emission is more significant and extensive. MAT and MAP have extremely significant positive correlation with soil NO_3_^-^ content, SOC and pH, and extremely significant negative correlation with TN. Therefore, it is necessary to consider the effects of climate factors, soil nitrogen content and soil physical and chemical properties on N_2_O emission.

## Discussion

### Analysis of the impact of nitrogen fertilizer application on nitrogen emissions

This study scrutinizes the complex relationship between nitrogen fertilizer application and nitrogen emissions. Nitrogen application is a major farm management practice that contributes 30–50% to crop yield [50]. While nitrogen application significantly enhances crop yields, its overuse poses ecological challenges and contributes substantially to agricultural GHG emissions [51, 52]. Increasing nitrogen fertilizer application (0–225 kg hm^-1^) increased N_2_O emission flux during rice-wheat cropping season according to Awais Shakoor *et al*. [53] Similarly, Williams K. Atakora *et al*. [54] found that at 60 kg N ha^-1^ yr^-1^, the N_2_O flux was significantly reduced and N_2_O emissions were reduced by 2–2.5 times compared to 120 kg N ha^-1^ yr^-1^. The results of this meta-analysis showed that under different conditions, the degree of influence of higher nitrogen application on the change of N_2_O emission increased by 1.33%-62.65% (Fig 8), and the mean change of emission was 25.75%. Overall, although the results are not very significant, they can promote the emission of N_2_O. The influence range of low nitrogen application on N_2_O emission change was -55.92% to 111.00% (Fig 6), and the average emission change was 9.13%, which still promoted N_2_O emission. On the whole, compared with the higher nitrogen application rate, the results are not significant, but under the influence of specific environmental conditions, N_2_O emission will be reduced. For example, under the condition that the soil texture is anthropogenic, the lower nitrogen application rate reduces the N_2_O emission by 55.92%. At MAT≥20°C and MAP≥1000mm, the N_2_O emission was reduced by 7.98% and 11.84%, respectively. The mean value of N_2_O emission change was 102.54% for the medium nitrogen application rate. Comparing the mean values of the three emission rates, it can be seen that the peak value of N_2_O emission appeared in the medium nitrogen application rate of 150–225 kg N ha^-1^.

In this meta-analysis, the effect size of nitrogen use efficiency under fertilizer rate <150 kg N ha^-1^ or ≥225 kg N ha^-1^ was higher than that under medium nitrogen rate (Fig 2), mainly because N_2_O emission was higher under nitrogen fertilizer rate of 150–225 kg N ha^-1^. However, excessive application of nitrogen fertilizer can cause serious ecological and environmental problems [51, 52]. Studies have shown that the greenhouse gases produced by the application of nitrogen fertilizer account for 36–52% of the total agricultural greenhouse gas emissions [55, 56]. Therefore, combining with environmental factors, the appropriate fertilizer amount can be allocated, and under the premise of reasonable control of N_2_O emission, it can better meet the demand for nitrogen in the growth process of crops. It can be seen that one of the ways to reasonably control N_2_O emission is to reasonably control the nitrogen application amount. However, the time and frequency of fertilization were not included in this study, which may lead to some uncertainties.

Lv *et al*. [57] reported data results showing that annual N_2_O emissions under organic fertilizer substitution conditions were lower than those under inorganic fertilizer alone over a 30-year simulation period. This result is consistent with the meta-analysis in this paper. Similar to previous studies, it can be seen from Figs 4 and 5 that under different environmental conditions, the application of inorganic fertilizer in farmland increased the N_2_O emission by 1.21% to 103.19%, with an average emission of 44.97%. The results are very significant. On the contrary, the N_2_O emission of organic fertilizers varied widely, ranging from -118.7%-157.63%, and the average emission was 14.47%. Under different environmental conditions, for example, the soil pH value is less than 6.5 (N_2_O emission reduced by 118.7%), MAT≥20°C (N_2_O emission reduced by 47.47%) and MAP≥1000mm (N_2_O emission reduced by 18.83%), the application of organic fertilizer will reduce N_2_O emission. Although it can be found from the mean value that the variation trend of N_2_O emission is still rising, it is still possible to artificially select specific environmental conditions to achieve the purpose of controlling N_2_O emission. A previous study also showed that [58], compared with conventional practice, common nitrogen fertilizer or urea combined with organic manure not only reduced the annual emission of N_2_O, but also reduced the direct emission factor. This is consistent with the conclusion of this paper. The potential of organic fertilizer to reduce N_2_O emission is greater than that of inorganic fertilizer application, which may be due to the higher nitrogen use efficiency in crop production.

### Analysis of impact of crop types on nitrogen emissions

Crop types in agricultural fields likewise affect N_2_O emissions. Guo *et al*. [59] carried out a data analysis and found that manure application relatively reduced N_2_O emissions in paddy fields compared to maize fields, whereas it significantly increased them in wheat fields. In addition, the average N_2_O emission coefficients of manure-applied wheat fields (mean: 0.36%) and maize fields (mean: 0.35%) were higher than those of rice fields (mean: 0.14%). Meanwhile, Zhou *et al*. [60] reported that the average N_2_O emission from rice is also lower than some other soils. Paddy fields are usually highly anaerobic, and most of the N_2_O produced after the application of nitrogen fertilizer is reduced to N_2_ (nitrogen gas) [61, 62]. This phenomenon was also confirmed by Garba ALIYU *et al*. [63] Similar to the former study, the results of this study show that as shown in Figs 9–11, the influence of paddy soil on N_2_O emission change can be significantly reduced by 38.84%, while wheat and maize fields can increase N_2_O emission by 71.97% and 268.24% at most. It can be concluded that among the three major crops, rice contributes the least to N_2_O emission. The mean variation of N_2_O emission in wheat and maize fields was 24.60% and 87.67%, respectively. From the data point of view, maize field has a greater impact on N_2_O emission. Liu *et al*. [64] also found that the overall average N_2_O emission flux in maize season is greater than that in wheat season, and the results are consistent with this meta-analysis.

Previous studies have reported that appropriate crop rotation cycles can significantly reduce N_2_O emissions [65]. However, the cycle of crop rotation was not specifically analyzed in this study, which may lead to some uncertainties, which may lead to different results. Ting Lan *et al*. [66] found that N_2_O emission was positively correlated with soil water content during wheat cultivation. In this meta-analysis, it is concluded that when MAP<1000mm, the study results are consistent, but when MAP≥1000mm, the degree of N_2_O emission change is minimal. Soil water content depends on soil texture, total rainfall, evapotranspiration and other factors, which significantly affect the production of N_2_O [67]. However, this paper did not analyze the factors affecting soil water such as evaporation and transpiration, so the results will be somewhat different from the former research. Therefore, in addition to fertilization type and fertilizer amount, crop type is also an important factor affecting N_2_O emission.

### N_2_O emission impact factors

Our analysis focused on the effects of experimental environmental conditions, such as different climatic factors, soil texture and soil physicochemical properties, on N_2_O emissions from agricultural fields to determine the specific extent of N_2_O emissions. Charles *et al*. [3] found that the application of organic fertilizer to fine textured soils reduced N_2_O emissions more than sandy soils. This is mainly due to the fact that the main process of N_2_O production in sandy soils undergoes nitrification [60]. Similarly, Ren, Sun *et al*. [36] found that the response of N_2_O emission to the application of organic fertilizer was significantly affected by soil texture and a negative correlation between N_2_O emission and soil clay content was obtained based on meta-analysis. The present meta-analysis showed that the higher the soil clay content, the lower the increase in N_2_O emissions, similar to the former study, when combined with the overall analysis. Previous report indicated that soil pH was the dominant variable in the differences in N_2_O emissions [68]. In addition, the trend of soil pH is different from our common knowledge that higher soil pH reduces N_2_O production by increasing the activity of N_2_O reductase during denitrification [69]. Regarding the relationship between soil pH and N_2_O emissions, the findings emphasize that the effect of soil pH on N_2_O emissions deserves further attention. Temperature is a key factor in nitrification-denitrification activities, and therefore is considered to have a significant effect on N_2_O emissions. SUN *et al*. [70] concluded that temperature was positively correlated with N_2_O emission, which was inconsistent with the meta-analysis of this paper, which may be related to external factors such as temperature zone, air temperature, precipitation, crop cultivation type and nitrogen application in the selected experimental site, and there will be some differences. Therefore, it is necessary to conduct more studies on the influencing factors to quantify their effects on N_2_O emissions in order to effectively develop management measures based on scientific evidence [37].

### Limitations and recommendations

Our analysis focused on the effects of climatic factors, soil physical and chemical properties fertilizer application and fertilizer type on N_2_O emissions from farmland, and these influences may also interact with each other. However, due to various factors such as different geographic locations and environmental conditions, differences in experimental design, and limitations in experimental conditions, the data related to some of the research results obtained are highly variable, and one cannot make good use of the regularity of the data to explore this. The present study integrates previously published results on the response of fertilizer applied to agricultural land to N_2_O emissions. Both the amount and the specific type of fertilizer applied can have an effect on N_2_O emissions from farmland where different crops are grown [3]. The specific nature of these fertilizers has rarely been described in previous studies, which simply categorized the fertilizers (type and amount of fertilizer applied), and therefore, there are limitations in analyzing the effects of different fertilizer applications on N_2_O emissions. Meanwhile, few studies have assessed the effect of nitrogen fertilizer on GHG emissions from the perspective of growth stage. Therefore, most of the articles selected for this study lacked theoretical and experimental data in this area, and the data that have been extracted are about total N_2_O emissions of the crop over the entire growth period or even over multiple growth periods.

In order to optimize the advantages of the three crop soils of rice, wheat and maize in terms of increasing agricultural productivity, reducing environmental losses and decreasing N_2_O emissions, it is necessary to develop a rational management practice that clarifies in detail the linkages between the proper application of organic fertilizers and inorganic nitrogen fertilizers, and N_2_O emissions.

## Conclusions

In this Meta-analysis, we investigated the effects of different fertilizer application methods on soil N_2_O emissions in agricultural fields, and it is known that the type of fertilizer is the main regulator of N_2_O emissions. Studies have shown that the application of organic fertilizer reduced N_2_O emission by 58.1%, while the application of inorganic fertilizer significantly increased N_2_O emission from farmland by 19.7%-101.05%. Organic fertilizer and inorganic nitrogen fertilizer should be reasonably matched, which is the best option for nitrogen application method to maximize the reduction of N_2_O emission, and to a certain extent, it also has a positive effect on the crop yield. Overall, N_2_O emissions from farmland are affected by fertilizer application, which is important for the green development of agriculture and the construction of a recycled agricultural green technology system.
